# Parents’ perceptions of the impact of COVID-19 and school transition on autistic children’s friendships

**DOI:** 10.1177/13623613221123734

**Published:** 2022-09-06

**Authors:** Laura Fox, Kathryn Asbury, Aimee Code, Umar Toseeb

**Affiliations:** University of York, UK

**Keywords:** autism, friendships, qualitative research, reflexive thematic analysis, school transition, special education

## Abstract

Friendships play a key role in supporting a successful transition to a new school for autistic children and young people. However, little is known about how these relationships have been impacted by restrictions put in place during the COVID-19 pandemic. This study aimed to explore how parents perceived the impact of COVID-19 on their autistic child’s friendships during transition to a new school. Semi-structured interviews were carried out with 14 parents of autistic children in the United Kingdom. Data were analysed using reflexive thematic analysis. Parents discussed a variety of experiences, which differed between and within school types and age groups. Several factors influenced children’s friendships during transition including support from their new school and others’ understanding of their needs. Parents described the differing expectations of what friendship was and how that affected their children’s existing and new relationships. For some, COVID-19 negatively impacted on friendships by reducing contact with existing and new friends. Others experienced positive or neutral experiences due to lockdown restrictions. This study highlights the diversity of needs among autistic children and calls for a personalised approach to transition support beyond the pandemic as one way of supporting autistic children to develop positive peer relationships.

**Lay abstract:**

Research shows that moving schools can be a challenging time for autistic children and young people. One factor that has been found to support successful transition is friendships. However, there is little research exploring how transition between schools affects autistic children’s friendships, and even less on how children’s relationships during transition have been impacted by COVID-19. Fourteen parents of autistic children and young people were interviewed about their child’s move to a new school and the impact they felt this had on their friendships. Parents described how moving with existing friends helped some children to find the transition less challenging. Others had differing experiences, with their children’s friendships playing a much smaller role in the move. Differences were also seen with regard to the impact of COVID-19, with some parents speaking of how hard being away from friends was for their child, while others found the social restrictions a welcome break from interacting with peers. The study highlights how different the experiences of autistic individuals, and their parents, can be and the importance of a child-centred approach to transition support.

Transitioning to a new school can be a major challenge for all children and young people (Bagnall et al., 2020), and particularly for autistic individuals (Canavan, 2014; Hannah & Topping, 2012, 2013). As children move to higher levels of education, such as secondary school, they are often required to navigate more complex environments, adjust to new academic and behavioural expectations, and handle changes in social interaction with peers (Anderson et al., 2000; Jindal-Snape & Miller, 2008; Symonds & Galton, 2014). Changes in peer relationships may be particularly problematic for autistic pupils, given their increased likelihood of experiencing social and communication challenges (American Psychiatric Association, 2013). Given the importance of peer relationships for successful transition (Peters, 2003), it is important to understand autistic peer relationships, and their implications, during transition to a new school.

Transitions can take place at any time in a child’s academic career. However, in the United Kingdom, they most commonly occur during the move from primary to secondary school, and from secondary to post-16 or post-18 education, or into the workplace. Schlossberg (2011) suggests that transitions can disrupt relationships, routine and roles, something which may be especially difficult for autistic students and their families. Transition theory helps us to understand why some individuals may react differently to the same type of transition and how successful transitions are dependent on the resources that individuals are provided with (Patton et al., 2016). This is especially important when supporting autistic students as they may have differing experiences of a transition to their neurotypical peers. Furthermore, research into Schlossberg’s theory has found that the children of parents who actively engage with the different phases of transition show higher rates of successful transition (McCoy, 2014). This model therefore highlights the importance of listening to and understanding parental experiences – as their children’s primary source of support in many cases – and the need to collaborate with parents during transition planning.

Friendships have been found to play an important protective role as children and young people navigate their social worlds, and in neurotypical children mutual friendships can be a source of social support and a protective factor against bullying (Brendgen & Poulin, 2018). Friendships have also been found to play a key role in supporting individuals during transitions. Having established friendships upon school entry has been found to help children to engage in conversation and play immediately, which in turn helps to establish positive school perceptions (Ladd, 1990). In the move from primary to secondary school, maintaining the same best friend during transition has been found to result in lower levels of conduct problems and higher academic achievement (Ng-Knight et al., 2019) and students maintaining a best friend in the move to University report being less lonely during their first year than peers who did not (Oswald & Clark, 2003). Research has also shown that making new friends may help to provide a more supportive learning environment, resulting in gains in school performance (Ladd, 1990). These findings highlight the importance of supporting individuals to maintain existing and make and maintain new friendships during and after educational transitions at all stages of development.

It is not uncommon for autistic individuals to have fewer friends or lower quality peer relationships than their non-autistic peers (Farley et al., 2009; Howlin et al., 2000; Shattuck et al., 2011). In a study including adolescents with additional needs, Shattuck et al. (2011) found that autistic adolescents were significantly less likely to be invited to social activities than adolescents with other special educational needs and disabilities (SENDs), and almost half reported never seeing their friends outside of school. This finding is supported by Kuo et al. (2013) who identified that autistic adolescents less frequently met their friends outside of school and were more likely to report fewer friendships compared with matched neurotypical peers. These findings suggests that a large proportion of autistic individuals may experience major obstacles to developing supportive and high-quality mutual friendships and being included in social engagements, which may affect their experience of educational transitions.

Autistic individuals may have different ideas of what friendship means compared with their classmates, often defining friendships as being about companionship more than affection and intimacy (Bauminger et al., 2004). Studies have shown that behaviours reflecting companionship, such as participating in common activities, are presented in autistic individuals’ descriptions of friendships (Daniel & Billingsley, 2010; Howard et al., 2006), traits which are often associated with younger neurotypical children (Newcomb & Bagwell, 1995). Despite these differing definitions of friendships, Calder et al. (2013) found that autistic children reported being satisfied with their friendships, even if those friendships had been rated by the children as lower quality, suggesting that autistic children can and do have friendships that they deem worthwhile.

Another factor to be considered is that not all autistic children transition into the same types of setting, or at the same time as their peers, impacting on their transition and the composition of their new peer group. For instance, while some transition from a mainstream primary school to a mainstream secondary school or college, others may transition to or from a special school or home education. As children generally favour friendships with those who are similar to themselves (homophily) (Bateman & Church, 2008; Hoffmann et al., 2021), the type of establishment a child attends could influence the number and quality of friendships available to them. Students in mainstream schools may find themselves in an environment predominantly occupied by neurotypical peers, whereas those in alternative provision (e.g. special schools) may have more opportunities for developing friendships with children who have similar needs. A recent systematic review showed that the prevalence of peer difficulties was much higher for autistic children in mainstream schools compared with those in special schools (Maiano et al., 2016). However, some gender differences have been identified with girls reporting more complex issues with peers in specialised settings compared with boys, who were more likely to experience bullying in mainstream settings (Cook et al., 2016, 2018). This suggests that friendships may differ not only between settings, but may also be influenced by gender.

In the period leading up to transition to secondary school (age 11 in England, Wales and Northern Ireland, age 12 in Scotland), autistic pupils have reported being worried about making new friendships and missing their primary school friends (Foulder-Hughes & Prior, 2014; Makin et al., 2017). They have also expressed concerns about ‘fitting in’ with peers at their new school (Foulder-Hughes & Prior, 2014), suggesting that the importance of – and challenges associated with – peer relationships during school transition is something that autistic pupils are aware of. After transition, children have reported that meeting other autistic children allowed them to benefit from shared experiences (Hannah & Topping, 2013) and allowed those that had struggled with peer relationships in primary school to form new friendships (Neal & Frederickson, 2016), supporting the homophily hypothesis. These positive experiences, however, are not echoed by all. Other autistic children have said that their social difficulties persisted into secondary school or college, and that they struggled to make or maintain friendships (Fortuna, 2014; Makin et al., 2017), with some reporting bullying (Fortuna, 2014; Neal & Frederickson, 2016).

Parents play an important role in ensuring that children are prepared and supported in their transition to a new school and can be a vital tool in successful transition planning (Defur et al., 2001; Kohler, 1999; Stoner et al., 2007). Parents may also be aware of, and ready to talk about, the experiences their child shares with them – or any observed changes in behaviour – before the child themselves. Furthermore, parents of autistic children have been found to provide a unique perspective on their children’s transitions, often one that is very different to the experiences discussed by parents of neurotypical children or those with other SENDs (Parsons et al., 2009). Listening to the experiences of parents and the knowledge they have acquired through supporting their children can therefore provide us with insight into how autistic children navigate their friendships during a transition to a new school. Furthermore, parents of autistic children have been found to be more concerned about their children’s peer relationships than parents of neurotypical children or those with other SENDs (Lindsay et al., 2016). This may mean that they pay particularly close attention to their child’s friendships, providing us with views that may not be obtainable via observations or pupil or teacher reports.

Peer relationships during school transition are an important challenge to address for autistic pupils in general. It is possible that this challenge may have been intensified by lockdowns linked to COVID-19 in 2020 and 2021. A series of lockdowns in the United Kingdom began in March 2020 with phased reopening of schools starting in June 2020 for children of key workers and those who were vulnerable, including autistic children (Bayrakdar & Guveli, 2020). Although the option to return to school was provided, research suggests that not all autistic pupils took up this opportunity, resulting in a large variety of educational experiences as provision was regularly interrupted by further lockdowns and self-isolation (Asbury et al., 2021). Therefore, some autistic pupils spent periods of their final year in their old establishment at home, as well as periods of their first year in their new school setting, and have missed a considerable amount of socialisation and schooling opportunities. Furthermore, students have had to undergo the additional transition of moving from school-based to home learning, significantly disrupting routines and the opportunity to develop new ones.

COVID-19 also disrupted the transition activities and support that schools would usually provide. Research has shown that a lack of consistency in the guidance and support provided to children and their parents during transition can create increased stress and anxiety (Doyle et al., 2017). Furthermore, routines in new schools were affected by the need to limit the risk of spreading the virus. Some examples include staggering the start of the school day, social distancing, mask-wearing and not moving around the school. It is feasible, therefore, that the impact of transition on autistic children’s peer relationships during COVID-19 differs from previous years, and this may provide new insights into how best to support autistic pupils through school transition.

Although there is research examining the importance of friendships during transition, there is a lack of research detailing parental views of how their autistic children’s friendships have been impacted during a time with social distancing measures in place. This study aimed to explore how parents perceived the impact of moving to a new school on their autistic children’s friendships during the COVID-19 pandemic. To develop a deeper understanding of these experiences two research questions were explored. First, how do parents perceive the impact of moving school during COVID-19 on their autistic children’s friendships? Second, to what extent do parents believe that COVID-19 has impacted their children’s friendships?

## Method

### Methodological approach

This study used reflexive thematic analysis (RTA), an interpretative approach to qualitative data analysis which aims to identify and analyse patterns or themes in a dataset (Braun & Clarke, 2021). Unlike some other forms of thematic analysis, RTA highlights the active role researchers play in the production of knowledge, and codes are acknowledged to represent the researcher’s interpretations of patterns of meaning across a dataset. RTA has been found to be well suited to studies aiming to amplify the voices of socially marginalised groups (Terry et al., 2017), such as autistic children and their families, and was therefore deemed the most suitable form of analysis for this study.

#### Ethics

The study was approved by the Education Ethics Committee at the University of York (Reference 20/05). Parents provided informed consent.

#### Participants

All of the 14 participants in the current UK-based study were parents or carers of school- or college-aged children with a formal diagnosis of Autism Spectrum Condition (ASC). They had all transitioned to a new educational setting during the COVID-19 pandemic, and all had an Education and Health Care Plan (EHCP), which means their need for additional support, and potentially to choose a special school over a mainstream school, is legally recognised. Participant information can be found in Table 1. All participants are referred to by a pseudonym to preserve anonymity. The sample was recruited from parents who had previously taken part in a study on the impact of COVID-19 on families with children who have a SEND who had agreed to be contacted about future studies (Asbury et al., 2021). Specific data on socioeconomic status and educational attainment levels were not recorded for this study.

**Table 1.** Child demographics.

Parent pseudonym	Child sex	Age	School year	Transition from	Transition to
Ella	M	7	3	Mainstream Primary	Mainstream Primary
Evelyn	M	10	6	Mainstream Primary	Special Primary
Bonnie	M	10	6	Special Primary	Special Primary
Sylvia	M	11	7	Mainstream Primary	Mainstream Secondary
Sammy	M	11	7	Mainstream Primary	Mainstream Secondary
Katie	M	11	7	Mainstream Primary	Special Secondary
Nicky	M	11	7	Special Primary	Special Secondary
Mollie	M	12	Not stated	Special Not stated	Home School
Maria	M	12	7	Mainstream Primary	Mainstream Secondary
Aishah	M	14	9	Special Secondary	Special Secondary
Erika	M	14	10	Mainstream Secondary	Special Secondary
Jenny	M	16	12	Special Secondary	Mainstream College
Lauren	F	16	11	Mainstream Secondary	Special Secondary
Lauren	M	18	13	Mainstream Secondary	Special College

Lauren participated in two separate interviews about her son and her daughter. The choice was made to keep Lauren’s pseudonym the same for both children as her experiences will be intertwined and therefore cannot be discussed as two completely separate experiences.

#### Data collection

Fourteen semi-structured interviews were carried out with parents of autistic children who had transitioned between educational settings during COVID-19. Participants were invited by email to take part in an online interview and asked to return a consent form to the researchers in advance. Interviews took part on a prearranged date via Zoom with one of the study authors (L.F. or A.C.). A semi-structured interview guide, developed by study author K.A., was used to shape the interview. The full interview schedule can be found in Appendix 1.

The duration of the interviews was, on average, 45 min, and all interviews were conducted over the course of 2 weeks in December 2020 via Zoom. Interviews were carried out at the end of the first term in the new school as it was hoped that, by then, any transient difficulties would have passed but the memory of transition would be recent. Interviews were recorded onto the University of York cloud and then transcribed verbatim. Verbatim transcriptions ensured that the intended meaning of participants’ accounts was not lost.

Participants were made aware prior to the interview that the interview would be recorded and were reminded of this at the beginning of the call. All participants were given the opportunity to ask questions about the study before and after the interview and were advised that they could stop at any time. On completing the interview participants were informed that they could request a copy of the transcript within 2 weeks of the interview taking place. After this time, all transcripts would be anonymised.

#### Analysis

Analysis was guided by the six-phase process suggested by Braun and Clarke (2021). Data analysis was carried out solely by the first author and began with the first author reading and re-reading the interview transcripts several times to be fully immersed in the data. Notes relating to analytic ideas or observations were made in relation to the individual data item and the dataset as a whole.

Codes were developed by systematically working through the entire dataset. Segments of data which were thought to be relevant or meaningful were identified and given code labels. Code labels were collated, and relevant segments of data were compiled for each code. Initial themes were generated by compiling codes which shared core concepts or ideas. Themes were then reviewed and refined, ensuring that they were built around a strong core concept, before being named. Throughout the whole process, the first author engaged with reflexivity via the use of a reflexive diary.

#### Community involvement and positionality

There was no community involvement in the design of the reported study.

Reflexive journaling was used by the first author, who conducted data coding and analysis, in order to reflect upon any personal views surrounding autism. As part of this reflexive process, it must be acknowledged that there are factors and experiences that influence the author’s view of autism. The first author’s understanding of autism is influenced by the Neurodiversity Movement. Within this model autism is viewed as a neurological difference which is a natural and valuable part of human variation. Therefore, the first author believes that professionals should advocate for social and environmental changes, which increase equity for autistic people, and it is possible that this perspective may have influenced the analysis. Furthermore, the first author has experience of working with autistic children in a mainstream secondary school, including supporting children during the transition into secondary, which may have made her particularly aware of the difficulties some autistic children face during school transition. While acknowledging this positionality, every effort was made to ensure the data were represented through the lens of the participants.

## Results

**Figure 1.**
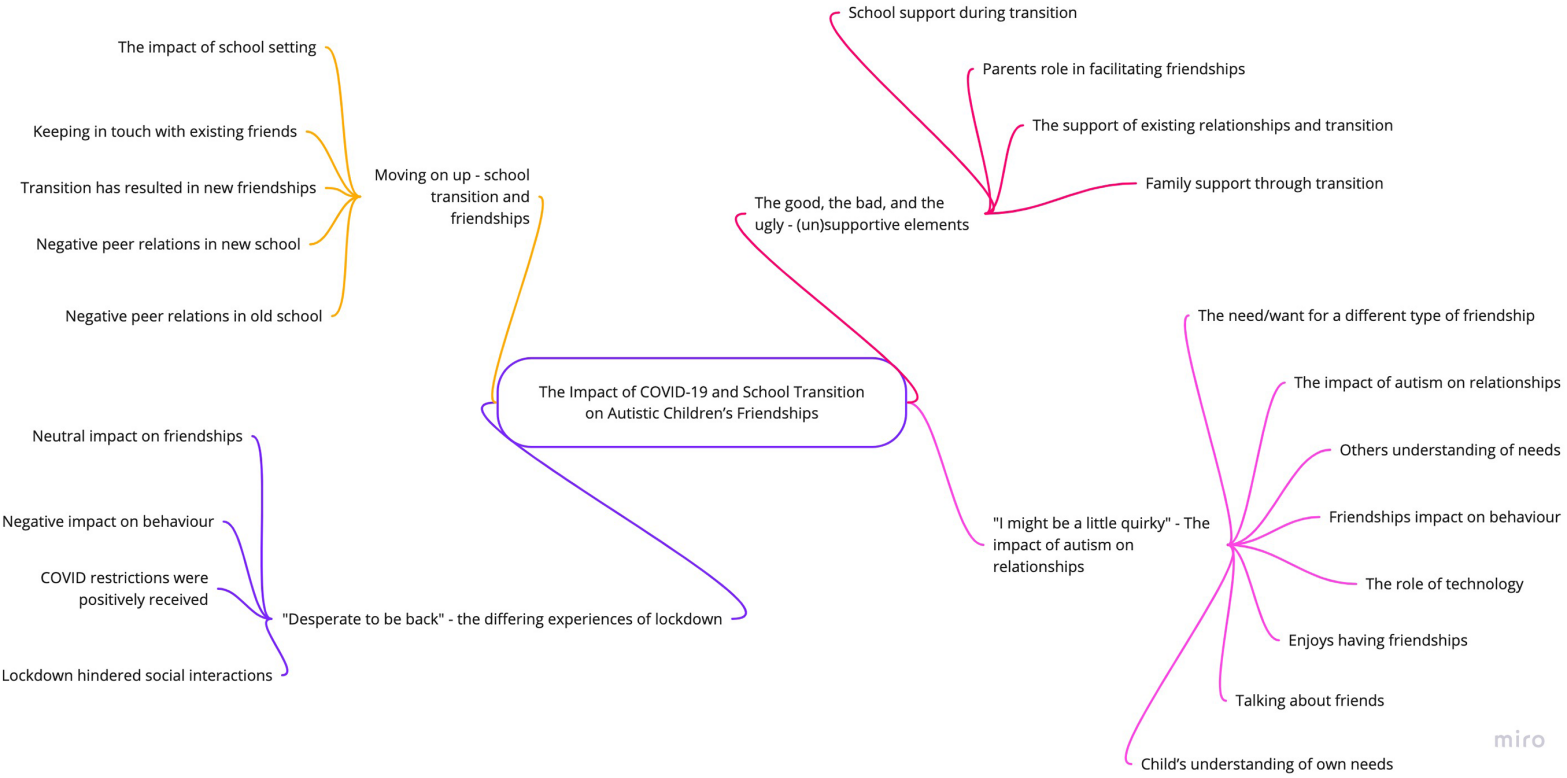
Thematic map.

Four themes were identified within the data: moving on up – school transition and friendships; the good, the bad, and the ugly – (un)supportive elements for transition; ‘I might be a little quirky’, the impact of autism on relationships; ‘Desperate to be back’, the differing experiences of lockdown. The four themes and their codes are presented in Figure 1.

### Moving on up – school transition and friendships

The impact that moving to a new school had on friendship was discussed by all participants, regardless of type of transition or age of child. Parents spoke about how their children’s friendships had changed as a result of transitioning to a new school and the impact a new school setting had on these relationships. Half of parents expressed that the move to a new school had helped their child to make new friends. For example, Maria described the relief she felt upon learning that her child had managed to make friends after the move to mainstream secondary school:

He made friends instantly which I think is great because that was my biggest worry because I know what he’s like he’s very ‘me’, he comes across as ‘what I say goes’ it’s ‘all about me and you have to do what I tell you to do’ so I was a bit worried.

Similarly, for Sammy, the move to a new school provided an opportunity for her son to move away from peer problems in his old school, which was seen as a positive experience: ‘it’s been fantastic because he’s been able to leave that crowd behind’.

However, for those with children transitioning from one special school setting to another, the development of new friendships appeared different. Only one parent mentioned, with uncertainty, that their child had made new friends: ‘I think he’s got some friends at school’ (Aishah), and others did not discuss the development of new friendships at all, speaking more of the peer interactions children had rather than friendships.

Keeping in touch with existing friends was an important factor during transition for many, and for some, friendships were maintained even when children had moved to different schools: ‘He’s kept in touch with some of his friends, there’s a couple of girls that were really smashing lasses’ (Sammy).

Lauren spoke of her experience of her daughter feeling extremely isolated when her friend transitioned to a new school before her:

She had one friend at mainstream and that friend actually left in December, so that’s when her attitude got worse because she had no one to talk to at all, she had no friends, she was isolating herself, she wouldn’t talk to anybody, and it got to the stage where I don’t think even the teachers could actually get anything out of her.

This was a common theme among the children and young people, and parents noted that some did not want to make new friends after the transition as their existing friends were all that they wanted: ‘he was like “no I don’t want to make new ones I’ve got the ones I’ve got and that’s all I want”’ (Erika).

The experiences above suggest that some parents believe that their children value friendships and they are missed when access is removed, highlighting the important role of friendships during transition. Parents whose children had transitioned from any setting into a special school discussed the impact they felt the school setting had on their children’s peer relationships more frequently than those attending a mainstream school, expressing that they believed children and staff were more understanding which allowed their children to gain confidence in interacting with peers:

They’re a lot more understanding because a lot of them have different issues of dyslexia or autism, so they’re kind of already more empathic than a general class of 30. So, it’s a nicer environment for him to be in. (Katie)

The interviews with these parents highlight how varied experiences can be across individuals and settings. Some parents expressed their relief that a new school had provided opportunities and suitable support for new friendships to blossom, while others discussed concern for the difficulties children were still having, even after the move.

### ‘I might be a little quirky’ – The impact of autism on relationships

The impact of being autistic on children’s friendships was raised by all parents. For some, the differing expectations of what their child thought friendship should be were seen as a barrier: ‘he’s not big on friendships. He doesn’t have typical friendships, I guess, being autistic [. . .] he’s not got that kind of bond with people, with his peers’ (Katie). For Nicky, the added stressors of her son bringing home friends from his special school setting for playdates also acted as a barrier: ‘playdates with SEN kids are, they’re difficult, because you’re doubling your stress levels by having two of them in the house’.

Nicky also described how her son’s needs, combined with attending a mainstream school, had impacted on his relationships with his peers and his academic work due to being removed from the classroom on a regular basis:

He was in mainstream for Key Stage 1 but it got to the point where he was spending more time outside of the classroom with his TA than he was with his peers, and so he wasn’t engaging in anything they were doing because he was the only one doing it.

Having peers who are understanding of their child’s needs was important for some parents, and this was more often expressed by those who had children attending mainstream schools. Parents said that having friends who were patient and understanding was beneficial to their child, and that knowing their child had friends that understood and shared their experience of having additional needs was a positive: ‘it’s lovely, because they all understand each other, and they just accept each other, and that’s what you need. You just need people to accept you for who you are, and they love you for who you are’ (Sammy).

For some parents, it was difficult to talk about how the transition had impacted their child’s new friendships as their children were reluctant to talk about their peers. Many children and young people only spoke about others in their new school to report feelings of negativity or disagreements: ‘yes but only in a negative way really, so yeah, I’d say only because things are going wrong’ (Bonnie). However, some children did openly discuss friendships with their parents who expressed that they enjoyed having the opportunity to be social.

For those children with existing or new friendships, parents noted that they chose to socialise with their friends through the use of technology and for some, this was a new form of socialising as a result of the pandemic: ‘We got him a phone in January this year so he’s been able to keep in touch digitally with his friends’ (Sammy).

Technology allowed one child to successfully socialise without the need to go out. His parent, Sylvia, spoke about this and her experience of the impact this had on her child’s social anxiety: ‘because [name] [is] quite reclusive. He doesn’t leave his bedroom really, he engages with his friends on social media, gaming, rather than going out, he’s got very severe social anxiety, so he doesn’t really leave the house’. Although two parents whose children attended special school did discuss technology, it was much more prominent among those attending mainstream schools, and older children tended to use gaming platforms and social media to interact with friends.

The differing needs of autistic individuals are reflected here in the varied experiences parents discussed in relation to how their children’s autism impacted peer relationships during the transition.

### ‘Desperate to be back’ – the differing experiences of lockdown

All parents described the impact that lockdown had on their children’s friendships and transition. For some, COVID-19 was perceived to have no impact on their children’s friendships: ‘he doesn’t have any contact with those children outside of school, so when we went into lockdown that wasn’t really any different for him’ (Evelyn).

Evelyn was not the only parent who found that social distancing and lockdown had little impact on their child’s interactions with friends. Sammy, a parent whose 11-year-old son transitioned from a mainstream primary to a mainstream secondary echoed Evelyn’s experience: ‘they didn’t see a lot of their friends outside of school anyway, so in that respect it hasn’t really had an impact’.

For others, lockdown negatively impacted their children’s ability to socialise, and parents described their children as desperate to see their friends and be back with peers: ‘he was so desperate to be back with his class [. . .] I think [he] just desperately missed them and was desperate to see his friends again’ (Bonnie).

The restrictions put in place not only hindered children’s ability to socialise, but in some cases had a negative impact on their behaviour. Children and young people were reported to be more insular, and parents expressed concern for how lockdown may impact on their child’s social skills: ‘I think the more the, the growing up bit, the social bit more than the work’ (Sylvia).

Although lockdown had negatively impacted some children’s friendships, positive changes in relation to how transitioning to a new school were being carried out were noted. Reduced contact with peers at school was thought to have enhanced many children’s move to a new school:

They’ve got staggered starts so he doesn’t see older children, same for lunch time and play time, he’s just with his year group and they don’t have assemblies, so all those sorts of things that he was scared about, [they] haven’t needed to be faced really. (Sylvia)

Although many challenging experiences were discussed in relation to the impact of COVID-19, it is clear that parents viewed some elements of social distancing as creating a positive environment for their autistic children by allowing them to interact with their peers in a less challenging environment.

### The good, the bad, and the ugly – (un)supportive elements

Having existing relationships helped support some children and young people in their move to a new school. For those children who transitioned with their existing friendship group, the move was perceived by parents to be much easier as a result of this: ‘we couldn’t have dreamt for an easier transition for him because he had all of his friends’ (Bonnie), suggesting that autistic children who move with a friend may experience more successful transitions.

Parents described how support from the new school also facilitated children’s transition by actively enabling individuals to engage with their peers. Maria spoke about how the school was supporting her son with his friendship skills and providing opportunities for him to be with peers through clubs: ‘the school itself have put a lot of support in place so he does like circle of friends; they’ve got like a science club they’ve got; little things that they can do at lunch time which keeps him going’, highlighting the importance of providing children with safe and supportive spaces in which they can engage with peers both during and outside of school hours.

Parents also discussed the role of family support. For Maria, she had provided support in the form of encouraging her child to mix with peers in drama and sports clubs, providing her child with skills to navigate meeting new people during transition:

Everyone’s like ‘well why have you done that?’ and I’m like ‘well he’s going to go into secondary school and he might not move up with all his friends’ [. . .] but if he knows about it now, it’ll make all the transitions a lot easier because if he knows that y’know, you mix and make new friends.

For others, support was found in a sibling experiencing the same transition: ‘he was fine because he was with his brother, they kind of use each other as support, and if he hadn’t had him, I don’t think he would have been as happy to have gone’ (Ella).

Support from existing relationships had facilitated a smoother transition for some children in both mainstream and specialised settings. Family support, however, was more commonly discussed by those whose children were attending mainstream school.

## Discussion

The findings of this study corroborate and expand upon previous research, supporting the important role that friendships can play in supporting transition, and the worries surrounding peer relationships shared by autistic children and their families. Parents in the current study discussed how transitioning to a new school was often supported by the presence of existing friendships and that, in the absence of familiar peers, support from the new school to encourage children to interact with new peers was well received. This has implications for parents in what to consider when choosing a school, and for schools in terms of how to support incoming autistic students. The wide range of experiences presented here highlights the differing needs of autistic individuals moving to a new school, as some children were reported as flourishing in their new environment, while others still endured peer difficulties. The added challenge of COVID-19 impacted many children’s ability to engage with their friends, but it was indicated by some parents that the social distancing measures schools adopted were beneficial to their autistic child in the move to a new school. Results will now be discussed by research question, followed by a concluding summary of the findings.

### Children’s friendships and transition

The move to a new school provided some children with the opportunity to leave old friendship groups and peer difficulties behind, something which was well received by parents. Parents talked about how being autistic was both a barrier and a facilitator during transition and how this impacted their children’s ability to make and maintain friendships. In line with previous literature, the ability to engage with new peers facilitated new friendships in some children that had struggled to have successful relationships previously (Neal & Frederickson, 2016). However, this was not the case for all, and some parents expressed concern for their children’s lack of friends, with transition disrupting previous relationships. This disruption to relationships and differing responses to transition is in line with transition theory and suggests a need for monitoring the wellbeing of individual autistic children following a major transition.

Although it was not possible for all children and young people to move with existing friends, for those that did, parents expressed that this was an important factor in a successful transition. Therefore, transition planning should aim to ensure that, if possible, autistic children are placed with an existing friend to help aid a successful transition. This may also be useful guidance to provide to parents at the point of choosing and applying to a new school.

For some, transition had negatively impacted on their children’s friendships. Children were said to have told their parents they were worried about losing existing friends, and the thought of changing friendship groups was concerning. This is in line with previous research finding that transition, and the changes that accompany it, is one of particular stress for autistic individuals (Makin et al., 2017). Parents also discussed the worry their children had about being able to ‘fit in’, similar to findings by Foulder-Hughes and Prior (2014). Therefore, creating an inclusive environment that helps to support children in building and maintaining friendships may be one of importance for autistic individuals after a move to a new educational setting.

In line with previous research, parents expressed that their children had differing expectations of what friendships should be. Calder et al. (2013) found that primary-aged autistic children could be successful in making and maintaining friendships, but that what children considered as a friendship was focused more on companionship than the sharing of emotions. This perspective was shared by parents in the current study who talked about how their children did not have ‘typical friendships’ and how when interacting with their peers they would enjoy playing games or interacting via gaming consoles. It could be argued that knowing about this want for companionship over a need for sharing emotions with others could influence the social interventions put in place for autistic children when supporting individuals in making successful friendships following a transition from or between primary settings. For example, social interventions could focus on providing safe spaces, such as gaming clubs, in which children can engage in parallel play with peers in which new relationships could be built over time. One parent named the ‘Circle of Friends’ initiative as a good example of this in her child’s school which has recently been found to foster peer acceptance (Schlieder et al., 2014).

Another finding was the need for others to have an understanding of their children’s needs. This was especially important if children were in mainstream school. It could be suggested that parents whose children were attending a mainstream setting were more aware of others’ understanding, given that their children were more likely to be friends with non-autistic peers than those attending a special setting. Educating the school community and raising awareness of autism may be an essential step in supporting autistic children to successfully build relationships after moving school (Kucharczyk et al., 2015).

Although parents supporting young people transitioning between or out of secondary school settings did share experiences with those moving from and between primary settings, there were some experiences specific to this age group.

Parents expressed their concerns for the lack of friends their child had and how this negatively impacted their child. It could be suggested that parents of older children are more aware of their child’s lack of friendships as at this age it is typical for adolescents to shift to relying more on peer relationships compared with family support (Lam et al., 2014). This may not be the case for autistic adolescents and studies have found that peer difficulties increase at this age, with peers being less understanding of their additional needs (Locke et al., 2010) which may result in parents being more conscious of their children’s friendship groups, or a lack of friends.

Unlike parents of younger children, those of adolescents did not discuss any positive impacts of lockdown. As older children were already accustomed to being in larger educational establishments the move into a new setting may have been less overwhelming compared with those moving from primary to secondary. It may also suggest that, as adolescents rely more heavily on their social network compared with younger children, the absence of friends during lockdown was more challenging for young people, suggesting that adolescents require more support if future lockdowns or restrictions are brought into place.

Another finding specific to those supporting older children in a special setting was the role parents played in facilitating their children’s friendships. Parents spoke of ‘getting kids together’ and how moving schools encouraged parents to be more proactive in ensuring their children met with friends. Support aimed at parents may be beneficial in helping them to find groups or activities that they can attend to support their children’s friendships.

Many experiences were shared at the group level; however, just as autistic individuals have heterogeneous needs, their parents’ experiences differed and appeared to be impacted by school setting. Those whose children were attending mainstream schools spoke in more detail about the importance of family support throughout the transition, and the role of technology in supporting existing and new friendships. These parents were also more likely to talk about experiences of difficulties in their children’s new school in comparison with those attending a special school, in line with previous studies (Maiano et al., 2016).

For parents whose children transitioned into a special school, the impact of school setting played a much more prominent role in supporting their children to make new friends, and they were more likely to have children whose friendships appeared to be unaffected by COVID-19. Parents of children attending special schools spoke more openly of the impact autistic characteristics, such as poor turn-taking, had on their children’s friendships. It could be suggested that these differing experiences may link to the level of needs children have. Those attending special school may have more complex needs than their peers attending mainstream, which may explain why parents were more aware of the impact their children’s needs had on making and maintaining friendships. Furthermore, children attending special settings often attend a school outside of their neighbourhood (Andrews, 2018), and those transitioning from a mainstream setting to a special setting may have been more prepared and better equipped with resources to deal with disruption to their friendship groups regardless of COVID-19, given that it would be unlikely that many of their peers from mainstream would be transitioning with them.

### The impact of COVID-19

One further finding of this present study was that COVID-19 had differing impacts on children’s friendships. Many parents reported that restrictions and lockdowns had negatively impacted their children’s ability to socialise both inside and outside of school. Children were reported to have missed friends over the time spent away from school, and many expressed the difficulties of being isolated with family members.

However, some elements of COVID-19 were seen as positive. Autistic individuals often have sensory difficulties, which can make moving around crowded schools or socialising with large groups difficult (Humphrey & Lewis, 2008). Changes implemented as a result of the pandemic, including secondary schools adopting a primary model and allowing children to remain in the same classroom throughout the day, were seen as beneficial as they reduced the number of peers children had to interact with throughout the school day. Extra time spent at home was also reported to improve family relationships, and parents commented that sibling relationships had improved and were used to support transition. Although not explicitly stated by parents, social distancing measure could also be seen as facilitating a more suitable environment for autistic children and young people to foster new friendships.

For others, no change was seen in their children’s friendships. As autistic children can often have differing expectations of what friendships are, parents expressed that prior to COVID-19 their children had not spent much time socialising outside of the house, and that friendships at school were often for school time only. This could suggest that providing more opportunities for children to socially engage with their peers in the school environment, such as after-school clubs or lunch clubs, may be one way to support autistic children to make and maintain friendships.

#### Limitations

One major limitation of the current study is that the voices and experiences of autistic children were not included. While we believe that parents’ experiences are an important angle on the issue of school transition it will undoubtedly be important to also explore this issue with children and young people, and potentially their teachers. This would be an excellent focus for future research. We have gained a rich insight into how a group of parents perceived their children’s friendships to have been impacted upon during transition to a new school in a global pandemic, but including autistic children – and their teachers – would further strengthen our understanding of the impact of transition on friendships and how to provide optimal support.

A further potential limitation is the wide age range of our participants’ children. For example, four of the five parents whose children moved from or between secondary settings had children attending special schools, which may have impacted on the findings. We have addressed this to some extent by considering the influence of child age and stage on parents’ experiences in this discussion. However, it would also be valuable for future research in this area to focus on parents of children at a similar age and stage (as well as on the children themselves and their teachers).

## Conclusion

This study demonstrates that the impact of COVID-19 on autistic children’s friendships during a move to a new school is complex. The findings suggest that autistic children can and do make friends after transitioning to a new school or college, and existing friendships can play a big role in supporting this transition. There were, however, differences not only between school setting and age, but among those whose children had had similar transitions.

The differing experiences presented here reinforce the need for a child-centred approach to transitions, with autistic children requiring more tailored and individual support than their non-autistic peers to ensure a successful transition (Fortuna, 2014). Nonetheless, this study – and indeed COVID-19 restrictions – offers some suggestions as to how autistic children might be supported when joining a new school. The study also highlights the benefits of employing qualitative methods in autism research to allow researchers to gain a deeper understanding of, in this case, parental perspectives of child friendships and how they can best support individuals.

It is also worth noting that some parents expressed that their children were often happy to play alone, and that as a result the COVID-19 pandemic had little impact on their friendships. These experiences highlight that although adults may feel compelled to encourage children to socialise with their peers, not all children will want or need the degree of social involvement that their non-autistic peers do. Parents comments’ within this study reflected the experiences expressed by autistic students in previous literature (Calder et al., 2013), raising the importance of listening to parents, as well as to their children, to gain further understanding of what support is needed to better support autistic children in making and maintaining friendships in a way that is in line with their needs.

Future work should expand upon the current findings by investigating how autistic children experience their friendships through further qualitative, in-depth approaches, to increase our understanding of the role friendships play during transition from the child’s perspective. Further exploratory work into how children experience friendships across different educational settings will also be beneficial for gaining a deeper understanding of the role school settings play in fostering autistic children’s friendships. Finally, research exploring how autistic children who transitioned during COVID-19 have adjusted to their new school now that social distancing measures have been relaxed would further expand our knowledge of factors that may support social interaction and transition for these individuals.

## Appendix 1

### School transitions during COVID-19

Thank you very much for agreeing to take part in our study. We are interviewing parents to find out how their child’s experience of moving to a new school or college has been affected by COVID19. The interview should last no more than 45 min and you are free to withdraw at any time. Do you have any questions before we get started?

1. Please can you tell me a bit about how things are going for your child [NAME] in their new school at the moment?
  (a) *What do they like or dislike about it?*
  (b) *How well would you say they have settled in?*
  (c) *What are the new arrangements for school drop-off and pick up (e.g. school transport if they use it) and how is that working out?*
2. How do you think your child [NAME] feels about their new school compared with their old school?
  (a) *When did they last see their old teachers and how did coronavirus and lockdown affect their relationships with their teachers?*
  (b) *Are they missing any particular teachers or Teaching Assistants?*
  (c) *How have they coped with the change in routine and the change in environment?*
  (d) *How are they coping with the learning in their new school?*
  (e) *Has this move to a new school altered your child’s behaviour in any way? Please can you tell me a little more about that.*
3. How have your child’s [NAME] friendships been affected by coronavirus and by the move to a new school?
  (a) *Can you tell me a bit about your child’s [NAME] friendships at their new school?*
  (b) *Who are their friends?*
  (c) *What do they tell you about their friends?*
  (d) *What do they say about the other children in their class*
  (e) *Does your child [NAME] still have contact with their old friends, and how do you think they feel about this? How can you tell (e.g. from what they say or from what they do)?*
4. Please describe the support that you and your child [NAME] received to prepare for the transition?
  (a) *What do you think about the quality of the support your family received?*
  (b) *Did the support come more from the old school or from the new school? Please can you tell me a little more about that?*
  (c) *What role did your child’s teacher or TA from their previous school play in supporting their transition?*
  (d) *How do you think the support you were offered was affected by the COVID pandemic?*
  (e) *What additional support would have been helpful to you or your child in making their transition smooth?*
5. In what ways do you think coronavirus has affected your child’s experience of moving to, and settling into, a new school? Please explain your answer.
  (a) *How has their learning been affected by being in lockdown before the move?*
  (b) *How has their behaviour been affected by being in lockdown before the move?*
  (c) *How have their relationships been affected by being in lockdown before the move?*
  (d) *Has your child been asked to self-isolate since starting their new school? If so, what has been the effect of that on them?*
6. How has home–school communication been?
  (a) *Have you had a parents evening yet? How did your child’s teacher say they were settling in?*
  (b) *What kind of communication have you had from your child’s teacher and/or school (other than a parent’s evening)?*
  (c) *How helpful has the SENDCo at the new school been?*
  (d) *To what extent does the new school value your input as a parent?*
  (e) *How does home-school communication compare to their old school?*
7. Thinking about the days before your child [NAME] started their new school, can you describe their behaviour and any thoughts they shared with you about the move.
8. Thinking about their very first day at their new school – please can you describe what happened before and after school?
9. Thinking about the return to school, after half term, please can you describe how your child [NAME] felt about going back, and how that compared to their first day in September?
10. Is there anything else you would like to tell us about your child’s [NAME] experience of moving school during the coronavirus pandemic?

Thank you so much for your time and for your thoughtful answers. They will really help us to build our understanding of school transition for children with Special Educational Needs, especially during a pandemic. If you would like to see a transcript of this interview, please email me on the address used to set up this interview within 2 weeks.

That’s all of my questions. Is there anything you would like to ask me before we finish?
